# Smell and Taste Impairments in Head and Neck Cancer Patients—A Scoping Review

**DOI:** 10.3390/nu17061087

**Published:** 2025-03-20

**Authors:** Nidhi Jha, Jed Speers, Lauren Gastineau, Shivani Patel, William Liu, Emily Pfahl, Apoorva Ramaswamy, Kai Zhao

**Affiliations:** Department of Otolaryngology, The Ohio State University, Columbus, OH 43210, USA; jed.speers@osumc.edu (J.S.); lauren.gastineau@osumc.edu (L.G.); shivani.patel@osumc.edu (S.P.); william.liu@osumc.edu (W.L.); emily.pfahl@osumc.edu (E.P.); apoorva.ramaswamy@osumc.edu (A.R.); zhao.1949@osu.edu (K.Z.)

**Keywords:** smell, taste, head and neck cancer

## Abstract

Head and neck cancer affects millions worldwide. The risk factors are numerous, including smoking, alcohol consumption, and human papillomavirus to name a few. While improved preventative, diagnostic, and treatment methods have decreased mortality rates, the treatments (chemotherapy, radiotherapy, or surgery) often result in smell and/or taste impairments. These can impact quality of life during and after cancer treatment. A scoping review was performed to understand current research and future directions regarding smell and taste impairments in head and neck cancer patients. PRISMA guidelines were followed and Rayyan.ai was used to search and compile journal articles. Three databases, EBSCOhost, Google Scholar, and PubMed, were also searched. Search terms included smell, taste, dysgeusia, ageusia, hypogeusia, parosmia, anosmia, hyposmia, dysosmia, and head and neck cancer. A total of 1580 articles were found through Rayyan.ai and 8022 were found through the three databases, which were manually screened. Articles assessing patients with a different malignancy, benign tumors, pediatric populations, animal studies, abstracts, and review articles were excluded. A total of 47 articles were found using this strategy. Of those we identified, 37 articles discussed taste impairments, 12 articles discussed smell impairments, and 3 articles discussed treatments for smell and/or taste impairments. All 37 articles concluded that there was some taste alteration in head and neck cancer patients due to their treatment. However, the specific taste qualities (sweet, sour, salty, or bitter) that were impaired, whether taste function returned to baseline, and which treatments led to impairments varied. For the 12 studies that assessed smell impairments, the results also varied. Some studies found significant objective impairments in smell while others found no significant impairment. Zinc sulfate was not found to be an effective treatment option for taste impairments; however, a liposomal spray showed some potential. Future studies should aim to understand which treatments and types of head and neck cancer lead to chemosensory impairments, whether chemosensory alterations negatively impact a patient’s nutritional status, and treatments or preventative measures for smell and taste changes.

## 1. Introduction

Nearly 1.5 million cases of head and neck cancer are diagnosed yearly [1]. Risk factors include smoking, alcohol consumption, human papillomavirus infection, Ebstein–Barr virus, betel nut consumption, and radiation exposure [2]. The initial symptoms patients experience depend on the location of the tumor; however, common symptoms include hoarseness, persistent sore throat, new neck mass, cranial nerve dysfunction, weight loss, chemosensory impairments (e.g., smell and taste), and difficulty chewing or swallowing [3,4]. The incidence of head and neck cancer has been increasing, yet improved preventative, diagnostic, and treatment methods have all decreased mortality rates worldwide [1]. Therefore, long-term outcomes and quality of life are of increasing importance in this population. Treatment options for these patients range from chemotherapy and radiotherapy to surgical removal of the tumor [5].

A common side effect of these treatments is sensory impairment, which can include losses in smell and/or taste [6,7,8,9,10,11]. These can negatively impact a patient’s quality of life and potentially lead to malnutrition during and after treatment [12,13,14]. Due to the SARS-CoV-2 pandemic, there has been an increase in research focused on smell and taste impairments. This has led to improvements in the diagnosis of chemosensory impairments as well as potential treatment options [15]. On one hand, recent studies have explored taste dysfunction in these patients; however, the results were inconsistent [8,10,11,16,17,18,19]. On the other hand, the impact of treatment on olfaction is less investigated. Furthermore, there is little information about how sensory impairments impact food consumption and nutritional status in patients. We performed a scoping review to understand the current research and future directions regarding smell and taste impairments in head and neck cancer patients.

## 2. Materials and Methods

PRISMA guidelines were followed for this scoping review [20]. Rayyan.ai was used for the search and compilation of journal articles [21,22,23]. Search terms included smell, taste, dysgeusia, ageusia, hypogeusia, parosmia, anosmia, hyposmia, dysosmia, and head and neck cancer to capture all available journal articles. Three additional databases (Google Scholar, EBSCOhost, and PubMed) were searched separately using the search terms listed above. A total of 1580 articles were found using Rayyan.ai, 7640 additional articles through Google Scholar, 372 articles through PubMed, and 10 through EBSCO-host.

An example of a PubMed search phrase is the following:

(“Smell”[tiab:~3] OR “anosmia”[tiab:~3] OR “hyposmia”[tiab:~3] OR “parosmia”[tiab:~3]) AND (“taste”[tiab:~3] OR “dysgeusia”[tiab:~3] OR “hypogeusia”[tiab:~3] OR “ageusia”[tiab:~3]) AND (“head and neck cancer”)

Relevant articles had to include measures to assess smell and/or taste impairment, or treatments for smell and/or taste impairment, or the nutritional impact of smell and/or taste impairment in head and neck cancer patients. Articles assessing patients with a different malignancy, benign tumors, pediatric populations, animal studies, abstracts, and review articles were excluded. Only studies assessing smell and taste objectively or subjectively in head and neck cancer patients were included.

**Figure 1:**
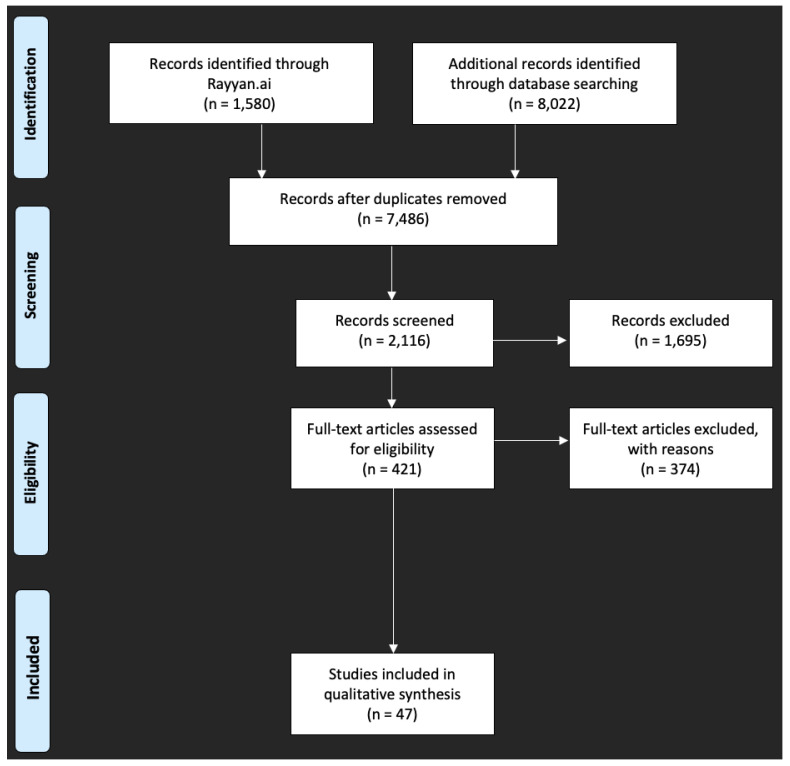
A PRISMA flow diagram detailing the journal article selection process.

Rayyan.ai allowed all six reviewers to independently assess titles and abstracts based on the inclusion and exclusion criteria. Reviewers were guided by the keywords listed above in order to identify relevant articles. Articles were rated as “included”, “excluded”, or “maybe” (i.e., insufficient information to decide eligibility) based on the title, then the abstract, and then the full-text reading. All articles listed as “included” and “maybe” were discussed as a group to decide eligibility. If there were discrepancies between reviewers, the article was discussed, and a final decision was made. Figure 1 is a PRISMA flow diagram that demonstrated the literature selection process. Papers included in this scoping review are organized in Table 1, which includes the following components: author, year published, patient population, sample size, measures used to assess chemosensory impairments, and results or findings.

## 3. Results

The search strategy aimed to gain a thorough understanding of smell and taste impairments in head and neck cancer patients. The journal articles included in this review spanned a variety of study designs and sample sizes, and used both objective and subjective assessment measures. Table 1 lists all the studies included in this scoping review.

### 3.1. Taste Impairments

A total of 37 articles assessed taste impairments in some degree; these included studies designed to assess the intensity of taste dysfunction, how changes in taste impacted quality of life, and the eating experience or nutritional impact of patients who experienced taste alterations. Patients received a range of treatments, including radiation therapy, chemotherapy, surgical removal of the tumor, or some combination of the three.

Studies used objective and/or subjective measures to assess changes in taste function. Some studies opted for objective measures using four taste qualities (sweet, sour, salty, and bitter). Seven studies focused specifically on assessing taste impairments at various dilutions, concentrations, or intensities of sweet, sour, salty, and bitter solutions. Other studies used subjective yet validated quality-of-life questionnaires such as the MD Anderson Symptom Inventory—Head and Neck Module, the European Organization for Research and Treatment of Cancer: Head and Neck Cancer Module-35, or the University of Washington Quality of Life version 4.

The median number of participants tested was 81 participants. Seven studies had a control group to compare the experimental group to. Patients were tested at multiple time points throughout treatment and after treatment.

All 37 studies found that there were significant changes in taste function among patients. In Riva et al., there were statistically significant, long-term taste impairments for the bitter and sour taste qualities between the control group and nasopharyngeal cancer (a subtype of head neck cancer) patient group [51]. There was also a statistically significant difference in the total taste scores between the control group and nasopharyngeal cancer patient group. Of the patients, 100% experienced bitter taste loss, 77.8% experienced salty taste loss, 70.4% experienced sour taste loss, and 40.7% experienced sweet taste loss. The sweet taste quality had the quickest recovery time while the bitter taste had the slowest recovery time. Nasopharyngeal cancer patients experienced maximum taste loss around the 4th week of chemoradiotherapy treatment [51]. Similarly, Mirza et al. found that patients had lower identification scores for bitter, salty, and sour tastes compared to the control group. They found that patients had a significant decrease in taste pore count from the first radiation treatment session to the second treatment session. Taste pore count in patients stabilized 6 months after radiation treatment was completed [44].

There are conflicting results on whether taste sensations return back to baseline after treatment. Stieb et al. reported that taste improved in the years after completing radiation, but plateaued after 5 years post-treatment [56]. Another study had similar results, stating that there is some improvement 1 year after intensity-modulated radiation therapy, but that taste did not return to baseline [49]. One study reported that while there were significant decreases in overall taste sensation during radiation treatment, all tastes returned to baseline after treatment [8]. All studies emphasized that taste alterations caused significant deteriorations in quality of life regardless of whether taste loss was partial or total.

Three studies focused on how treating head and neck cancer surgically impacted taste function [18,25,40]. Out of these three, one study compared transoral robotic surgery to definitive radiation treatment. Two of these studies utilized the University of Washington Quality of Life Questionnaire, while one study had patients complete posterolateral swab taste testing or whole-mouth taste testing 2 weeks after surgery [18,25,40]. In all these studies, patients reported their taste function was impacted post-operatively. One study showed that regional swab taste identification was decreased after surgery on the side of the tumor [18]. When comparing transoral robotic surgery to radiation, patients who underwent transoral robotic surgery scored significantly better in the saliva and taste domain than patients undergoing definitive radiation at all four time points. Among transoral robotic surgery patients, adjuvant chemotherapy was associated with worse quality-of-life scores in the taste domain at 6 and 12 months post-operatively [40].

One study evaluated the impact of radiation dose and salivary output on taste dysfunction [50]. Radiation dose to the taste bud bearing tongue mucosa was not significantly correlated with taste impairment [58]. However, there was a significant correlation between radiation dosage to the submandibular and parotid gland on the side of the tumor and moderate-to-severe taste impairment [55].

Four studies focused on how taste dysfunction impacted the nutritional status in patients. In Li et al., 67% of patients experienced significant weight loss [12]. Changes in taste functions were also associated with malnutrition, with malnutrition prevalence being significantly higher at the 3-month follow-up than the 6-month follow-up [13]. Finally, there was a statistically significant increase in patients who experienced taste loss in the food-restriction group [27]. Pingili et al. found that 40.2% of oral and oropharyngeal cancer patients experienced malnutrition [13]. They also found that malnutrition was significantly lower in patients who had taken nutritional supplements [13].

For patients who are unable to consume enough food, have difficulty swallowing, or struggle with severe nausea, a feeding tube is necessary to prevent malnutrition and continue treatment. One study focused on how taste alterations impacted quality of life in tube-fed vs. orally fed head and neck cancer patients receiving radiotherapy, chemotherapy, or a combination of the two [7]. All tube-fed patients had oral intake in the form of clear liquids and supplements. As the study progressed, the most distressing symptoms for tube-fed patients and orally fed patients were swallowing and saliva, respectively [7]. Taste was also considered an important symptom for both patient groups; however, it was not the most distressing symptom [7]. Tube-fed patients had significantly higher Taste Complaint Scores than orally fed patients [7]. At the 2.5-month follow-up, 49.3% of orally fed patients and 62.1% of tube-fed patients had significant taste impairments according to the UW-QoL assessment [7].

### 3.2. Smell Impairments

A total of 12 articles discussed smell impairments in head and neck cancer patients who received a range of treatments. These treatments include chemotherapy, radiotherapy, surgical removal of the tumor, or a combination of the three. The median number of study participants was 42, with three studies including a healthy control group. Studies used either objective methods or subjective methods to assess the association between head and neck cancer treatment and olfactory impairment. Objective measures included Sniffin’ sticks to assess odor threshold, discrimination, and identification, the NIH Toolbox Odor Identification Test, or the University of Pennsylvania Smell Identification Test. Subjective measures included the quality-of-life questionnaires previously listed above.

The findings of the 12 studies were limited and conflicting. Barbosa Da Silva et al. found that head and neck cancer patients had significantly worse identification performance for 33 out of the 40 odorants compared to the control group prior to treatment [24]. The chance of a patient having olfactory losses was 10 times higher than in the control group [24]. However, this study focused on symptoms before beginning treatment rather than during or after treatment [24]. Another study reported that smell function significantly worsened in the long term according to the Health-Related Quality of Life Questionnaire [48]. Other studies found significant subjective differences between the patient and control groups in regard to olfactory impairment [29,51,53]. In these studies, up to 92% of patients reported some degree of smell alteration and 51.1% of patients reported severe alterations in smell [38]. Yet these were subjective findings the patients reported rather than objective findings through validated olfactory assessments. Veyseller et al. reported that there was a significant difference between olfactory scores between nasopharyngeal cancer patients and the control group. Olfactory bulb volumes of the healthy control group were significantly higher than in the nasopharyngeal cancer patient group [57].

Unlike taste, there is limited information on how smell impairment impacts nutrition and quality of life in head and neck cancer patients. Only one study indicated that smell alterations were significantly correlated with anxiety and quality-of-life survival scores [38].

### 3.3. Treatments for Sensory Impairments

Treatments for head and neck patients experiencing smell and/or taste impairments are limited. One preventative option is to limit radiation to the taste bud bearing tongue mucosa and the olfactory bulb [57]. Another potential option is to decrease the treatment dose to protect the taste bud bearing tongue mucosa and the olfactory bulb. Both of these preventative options are difficult, since radiation doses reach large quantities and it is difficult to localize treatments for all head and neck cancers, especially if they have metastasized.

Three articles were found that discussed two potential treatments for smell and taste impairments in head and neck cancer patients: zinc sulfate and a liposomal spray. The number of participants in these three studies was 35, 98, and 169, respectively. Two of the studies were randomized control trials while the third was a pre–post study [31,33,45]. Zinc sulfate was only assessed as a potential treatment for taste impairment while the liposomal spray was tested as a potential treatment for both taste and smell.

Zinc sulfate was assigned three times a day throughout radiation and 1-month post-treatment for experimental group patients. Control group patients were given a placebo. All patients had taste impairments during the first two months of treatment. In this particular study, zinc sulfate did not favorably affect taste recovery [31]. In another study, zinc sulfate was given to prevent the effects of taste alteration in the experimental group. In patients who received zinc, taste perception threshold did not change at the end of radiation except for the sour taste. In the placebo group, taste perception threshold significantly increased for all tastes. In the zinc group, there was only a slight increase in the perception threshold for the salty taste post-treatment [45].

The liposomal spray was used for the nose and mouth for 2 months. All participants suffered from a smell and taste disorder after finishing head and neck cancer treatment. Patients with primary radiation had a decreased sense of smell; however, this was not statistically significant. Patients underwent a variety of treatments and were split into three separate groups: only surgery, surgery and adjuvant radiochemotherapy, and primarily radiochemotherapy. Patients were requested to take the liposomal spray for 2 months, three times a day, with five sprays in each nostril and the mouth [33]. After liposomal application, sense of smell increased significantly for all three groups. The same results were seen for taste, with significant increases being seen in all three groups. Of the patients, 13.3% showed no improvement for smell, with their results showing no greater than a 2-point increase in their TDI scores. Meanwhile, 10.2% of patients showed no improvement in taste, with their results showing no greater than a 1-point increase in their taste strip score [33]. These results indicate a potential treatment option for patients who experience smell and taste impairments.

## 4. Discussion

This scoping review identified and reported on 47 articles and publications discussing smell and taste alterations in head and neck cancer patients. All 37 articles about taste impairments reported significant changes in taste function. There was conflicting information about which taste qualities were the most impacted by head and neck cancer treatment and if taste function returned to baseline after treatment was completed. Since the information on smell alterations was limited, conclusive results could not be determined. Three studies found significant impairments in olfactory function for head and neck cancer patients. Only 9 out of 47 studies had a control group. Not all studies used objective measures to assess smell and taste losses in patients. Some studies used quality-of-life questionnaires that inquired about smell and taste dysfunction.

This scoping review also reported on how surgical interventions for head and neck cancer impacted taste function. Three studies focused on the impact of surgical interventions on chemosensory function. These studies found that taste function was impacted post-operatively. Transoral robotic surgery patients had better outcomes for the saliva and taste domain than patients undergoing definitive radiation, but had worse quality-of-life scores in the taste domain if they received adjuvant chemotherapy [40]. Another study reported that taste identification was decreased on the side of the tumor post-operatively [18]. This indicates that surgical interventions could have a negative effect on chemosensory function.

New surgical interventions could be a possible treatment to alleviate smell and taste impairments. Menzel et al. studied various endoscopic approaches to electrically stimulate the olfactory bulb in human cadavers. This study suggests that this surgical approach is low-to-medium risk for the patient and allows the electrode to be near the olfactory bulb [59]. Similarly, electrical stimulation could improve the function of certain taste receptors. Katsuki et al. studied how electrical taste stimulation impacted salt taste perception in stroke patients [60]. Their findings suggested that salt taste perception was significantly enhanced in stroke patients without significantly altering the salt concentration. Funamizu et al. conducted a similar study to understand how transcutaneous electrical stimulation near the mouth impacted taste perception [61]. Similarly to Katsuki et al., the intensity of the salty taste was enhanced along with the overall taste of six different foods [61]. While further research is necessary in the head and neck cancer population, these preliminary studies showed promising results to enhance taste perception using a non-invasive method.

The current scoping review captured research on how head and neck cancer impacts smell and taste. Recent advances have allowed for personalized and localized treatment options for patients. These could lead to new chemosensory side effects that have not previously been studied. Moreover, since treatment options vary depending on the type of head and neck cancer, chemosensory alterations could differ based on the treatment a patient receives.

Future research should aim to understand what specifically is causing chemosensory impairments in head and neck cancer patients receiving treatment. They should also inquire about which types of head and neck cancer treatments are more likely to cause smell and/or taste impairments. The nutritional status of head and neck cancer patients, specifically how they navigate consuming food, is an important aspect of treatment that should also be followed. Finally, potential treatments should be investigated. Right now, only three studies have assessed potential treatments: zinc sulfate and a liposomal spray. It would be beneficial to continue evaluating potential treatments for smell and taste alterations due to head and neck cancer treatments, so patients have multiple methods to choose from.

## Figures and Tables

**Table 1:** Studies on taste and smell alterations in head and neck cancer patients.

Author	Year	Patient Population	Sample Size	Measures Used	Results
Alfaro et al. [6]	2021	Head and neck cancer survivors	40 patients; 20 controls	UPSIT for smell function Label Magnitude Scale for regional/whole-mouth taste perception	Survivors were less likely to identify lower concentrations of bitter, sweet, and salty stimuli in the tongue tip.
Alvarez-Camacho et al. [7]	2016	Head and neck cancer patients who were both tube-fed and orally fed	85 patients	UWQoL questionnaire V3 Chemosensory Complaint Score (CCS)	Both survey scores decreased for each patient population. The scores increased after treatment but did not return to pre-treatment levels. CCS scores differed between the two groups at every time point and were a significant predictor of QoL.
Asif et al. [8]	2020	Head and neck cancer patients treated with radiation therapy	21 patients	Validated taste strips EORTC-QLQ-H&N35	There were significant decreases in overall taste sensations between the three time points. Significant decreases in sensation of sweet and salty tastes. All tastes returned to baseline at the third time point onwards.
Baharvand et al. [16]	2013	Head and neck cancer patients	22 patients	Whole-mouth technique EORTC-QLQ-H&N35	Of the patients, 72.2% had total taste loss and all patients had some degree of dysgeusia. Significant changes were observed in concentrations of different tastes before and after radiotherapy. Impairment was observed in mainly salty and bitter tastes.
Barbosa Da Silva et al. [24]	2023	Head and neck cancer patients	31 patients; 31 controls	UPSIT VAS	UPSIT score was significantly lower in head and neck cancer patients compared to the control group. Patients were 10 times more likely to have olfactory losses than the control group. Patients had worse scent identification scores.
Biazevic et al. [25]	2008	Squamous cell carcinoma of the head and neck	47 patients	UWQoL	Patients completed the questionnaire pre- and post-operatively. Taste was one of the most affected domains post-operatively.
Brown et al. [26]	2017	Head and neck cancer patients	125 patients	Observational study	Both groups experienced similar levels of weight loss. The most common reasons for non-adherence were loss of taste, early satiety, and nausea. Loss of taste became the primary reason for non-adherence in weeks 4–7.
Chen et al. [9]	2022	Head and neck cancer patients	87 patients	Whole-mouth taste solutions Subjective total taste acuity EORTC-QLQ-H&N35	Before radiation, all patients had normal taste function. Most patient experienced taste disturbance 3 months after treatment. There was a positive correlation between subject taste loss and objectively measured taste loss in all four taste qualities. Recovery of the four taste qualities was observed at 3 months and 6 months post-treatment.
Chen et al. [10]	2019	Head and neck cancer patients receiving intensity-modulated radiotherapy	88 patients	EORTC-QLQ-H&N35	Glossectomy most significantly predicted taste impairments. The mean radiation dose was borderline significant in head and neck cancer patients.
Cruz et al. [27]	2012	Oral and oropharyngeal cancer patients	120 patients	Structured food frequency questionnaire	Of the patients, 33% suffered from major food restrictions and 40% suffered from mild food restrictions. Taste loss, large tumor size, tooth loss, and lymph node metastasis were associated with food restrictions.
Dalton et al. [28]	2022	Head and neck cancer survivors	4 patients	Case series	All individuals reported sensory function changes impacting their ability to taste and their desire to eat. Taste alterations were experienced by all patients. Taste intensity and pleasantness were altered. Weight loss was present in all cases.
Epstein et al. [29]	2020	Head and neck cancer patients	10 patients	NCI common terminology criteria for adverse events V4 Scale of subjective total taste acuity	Spicy and pungent perception was the most strongly disliked testing stimuli and the most intense. Bitter taste intensity was weak during treatment but was very strong following treatment. Umami and fat taste perception had the highest intensity during treatment. Changes in smell function were limited to three patients during treatment but improved after treatment.
Gurushekar et al. [30]	2020	Head and neck cancer patients who underwent radiotherapy	34 patients	Italian Nose Obstruction Symptom Evaluation Odor identification test	Olfactory identification score, olfactory threshold score, and median combined olfactory score showed a significant decrease at the end of radiation therapy. There was significant improvement in the 3-month follow-up period but olfactory function did not return to baseline.
Halyard et al. [31]	2007	Head and neck cancer patients undergoing radiotherapy	169 patients	Experimental group was assigned zinc sulfate 45 mg orally three times a day throughout radiation and 1 month post-radiation Wickham questionnaire	Zinc sulfate did not favorably affect taste recovery.
Haxel et al. [32]	2016	Advanced squamous cell carcinoma of the head and neck	33 patients	Sniffin’ sticks	The mean decrease in TDI score was 0.72, 2.1, and 0.77 for the first, second, and third cycles, respectively. The decrease between the first and second cycles was significant. The olfactory threshold consecutively decreased during all three cycles.
Heiser et al. [33]	2016	Head and neck cancer patients	98 patients	Sniffin’ sticks Taste strips Visual Analog Scale	All patients suffered from a smell and taste disorder after finishing treatment. Smell and taste function significantly increased after usage of the liposomal spray in all three groups.
Jalali et al. [34]	2014	Head and neck patients receiving radiotherapy	54 patients	48 sniff bottles using the TLD system 16 bottles contained n-butanol 32 remaining bottles were blanks	Mean olfactory threshold scores significantly decreased at various time points after radiotherapy. Olfactory threshold was significantly decreased 2–6 weeks after radiotherapy initiation.
Jin et al. [35]	2018	Head and neck patients receiving radiotherapy	114 patients	Single-item taste assessment to evaluate intensity and severity CiTAS to assess chemotherapy-induced taste changes	Prevalence of subjective taste alteration and perceived interference with dietary intake increased from baseline to post-treatment. There were significant decreases in BMI from baseline to mid-treatment and mid-treatment to post-treatment. Subjective taste alterations were a persistent symptom among patients during and after treatment.
Jun et al. [36]	2023	Oropharyngeal cancer patients treated with radiotherapy or chemoradiation	33 patients	EORTC QoL-C30 CT scan of the head and neck region	Mean score for the taste disorder portion of the questionnaire increased and was significantly correlated with the parotid gland volume decreasing.
Kamstra et al. [37]	2011	Oral and oropharyngeal cancer patients	89 patients	Oral symptom assessment Mandibular function impairment questionnaire	A majority of patients had abnormal taste function. Lack of saliva could be the cause of taste disturbances.
Li et al. [12]	2023	Head and neck cancer patients who had received radiotherapy	94 patients	Head and neck patient symptom checklist	During radiotherapy, the number of nutrition impact symptoms gradually increased. Taste changes were experienced at a high intensity. Of the patients, 67% experienced weight loss during the study.
Liang et al. [38]	2024	Nasopharyngeal carcinoma patients	135 patients	Taste and smell survey	Of the patients, 91.1% reported taste and smell alterations, and 51.1% of patients had severe alterations. Olfactory sensitivity changes were reported in 48.9% of patients. These alterations were significantly correlated with anxiety and quality-of-life survival scores.
Lilja et al. [39]	2018	Head and neck cancer patients	44 patients	Electrogustometry UWQoL questionnaire	All post-treatment values were significantly higher than pre-treatment. Higher scores in odor detection values were observed in the 6-week and 3-month post-operative tests compared with pre-operative values for the tumor side. Electrogustometry values for taste on the tumor side were significantly impaired at 5 weeks and 3 months compared to pre-operative results.
Ling et al. [40]	2016	Oropharyngeal cancer patients treated with transoral robotic surgery (TORS) or chemoradiotherapy (CRT)	92 patients	UWQoL V4	TORS patients who received adjuvant chemotherapy had a worse quality-of-life score in the taste domain at 6 and 12 months compared to patients who underwent TORS only.
Manojan et al. [41]	2024	Head and neck cancer patients excluding malignancies of the nasopharynx and nose	34 patients	Italian Nose Symptom Evaluation Odor identification with 10 common odorants	Olfactory identification score, olfactory threshold score, and median combined olfactory score showed significant decreases at the end of radiation therapy. There was significant but incomplete recovery in the 3-month follow-up period.
Manzar et al. [42]	2020	Oropharyngeal cancer patients receiving intensity-modulated proton therapy (IMPT) or volumetric-modulated arc therapy (VMAT)	46 IMPT patients; 259 VMAT patients	EORTC-QLQ-H&N35 Provider assessed toxicities (CTCAE v4.03)	Patients reported significantly increased dysgeusia with IMPT compared to VMAT. However, IMPT was associated with significantly lower PEG-tube placement and significantly less hospitalizations 60 days post-treatment compared to VMAT.
Mathlin et al. [11]	2023	Head and neck cancer patients	61 patients	MDASI-HN questionnaire Supplementary questionnaire for patients with dysgeusia in week 4	At week 1, 30% of participants reported taste changes. At week 4, 97% of participants reported taste changes. Participants with a diagnosis of oropharyngeal cancer were most likely to report moderate or severe dysgeusia (28/32). Of the chemotherapy patients, 88% reported dysgeusia compared to 64% of radiotherapy patients.
Mau-Sun, H et al. [43]	1999	Nasopharyngeal carcinoma patients	24 patients pre-radiotherapy, 25 patients during radiotherapy, and 36 controls	Olfactory function battery test	Nasopharyngeal carcinoma patients had olfactory processing impairments. This included absolute thresholds, odor cross-matching, verbal identification of odors, and recall identification of odors.
Mclaughin [17]	2013	Head and neck cancer patients	98 patients	Taste discrimination testing with high, medium, and low concentrations of sweet-, salty-, sour-, and bitter-tasting solutions	Of the patients, 92.4% had some measurable taste dysfunction. There was confusion between the bitter and sour taste solutions. There was difficulty discriminating between the concentrations of the sweet solution. There was statistically significant weight loss associated with dysgeusia.
Mirza et al. [44]	2008	Head and neck cancer patients	8 patients; 17 controls	4 suprathreshold stimulants that represented sweet, sour, salty, and bitter tastes	Patients had lower taste identification scores for the bitter, salty, and sour tastes. Taste pores were decreased in the irradiated group. There was a significant decrease in taste pore count from the first session to the second session. This stabilized by 6 months.
Mulasi et al. [14]	2020	Advanced head and neck cancer patients	19 patients	Scored Patient-Generated Subjective Global Assessment to evaluate nutritional status EORTC QLQ-30 and QLQ-H&N35	Patients reported more severe problems with taste and smell sensations at their 1-month follow-up. Smell and taste slightly improved 3 months post-treatment but did not return to baseline. The largest decline in body weight was found 1 month after the treatment period. Body weight showed improvement at 3 months post-treatment but patients did not return to baseline.
Najafizade et al. [45]	2013	Head and neck cancer patients who received radiotherapy	35 patients	Detection and recognition thresholds for four taste qualities Randomized, placebo-controlled trial	In patients who received zinc, taste perception threshold did not change at the end of radiation except for the sour taste. One month after treatment completion, taste perception threshold was significantly increased in the placebo group for all tastes. In the zinc group, there was only a slight increase in the perception threshold for the salty taste.
Negi et al. [46]	2017	Head and neck cancer patients	27 patients	Taste recognition using sweet, sour, salty, and bitter tastes	All 100% of patients had maximum taste loss for the bitter taste during the 7th week, 77.8% of patients had maximum taste loss for the salty taste, 70.4% of patients had maximum taste loss for the sour taste, and 40.7% of patients had maximum taste loss for the sweet taste. Maximum taste loss was most pronounced at the 4th week.
Ogama et al. [47]	2010	Head and neck cancer patients receiving radiation therapy	208 patients	Appetite questionnaire 48 items focused on dietary preferences	The overall ease of consuming a meal increases as smooth food form, chewable texture, and suitable texture increase. This results in an enhanced gustatory sensitivity and olfaction without intensifying pain.
Oskam et al. [48]	2013	Oral and oropharyngeal cancer survivors	80 patients	EORTC-QLQ-C30 EORTC-QLQ-H&N35 HRQoL questionnaire EAT-10	Taste and smell significantly worsened in the long term according to the HRQoL questionnaire at 2 years. Of the patients, 62% were on a special diet at the time of treatment. Continued improvement was seen from 1 to 2 years. EAT-10 scores returned to baseline by 2 years.
Pearstein et al. [49]	2019	HPV-associated oropharyngeal carcinoma patients	126 patients	EORTC-QLQ-C30 EORTC-QLQ-H&N35 EAT-10	Taste and other senses did not return to baseline levels. There was some improvement in taste after 1 year.
Pingili et al. [13]	2021	Oral and oropharyngeal cancer patients	97 patients	EORTC-QLQ Head and neck mandibular function impairment questionnaire	Sensory difficulty for taste sensation was significantly associated with malnutrition. The prevalence of malnutrition was significantly higher at 3 months compared to 6 months.
Riantiningtyas et al. [50]	2023	Head and neck cancer patients	30 patients; 30 controls	Whole-mouth chemesthetic stimulation with menthol and capsaicin Food texture discrimination Temperature discrimination	Head and neck cancer patients demonstrated significantly lower chemesthetic sensitivity for medium and high concentrations. Patients were less sensitive to food textures and had lower tactile sensitivity.
Riva et al. [51]	2015	Nasopharyngeal cancer patients	30 patients; 30 controls	Sniffin’ sticks Taste strips with sweet, sour, salty, and bitter solutions	A statistically significant difference between healthy subjects and patients was seen. Chemoradiotherapy for nasopharyngeal cancer patients induced long-term smell and taste impairments. There were no significant differences in olfactory function when comparing different radiation techniques. There were statistically significant differences for the sweet, bitter, sour, and salty taste strips when comparing different radiation techniques. There was a statistically significant difference between the bitter, sour, and total taste scores between the control and treatment groups.
Rogers et al. [52]	2010	Oral and oropharyngeal cancer patients	250 patients	UWQoL questionnaire V4 Xerostomia-related QoL scale (XeQoLS)	There was a significant correlation between the XeQoLS score with the UWQoL domain scores. There was a negative correlation between xerostomia and taste.
Sandow et al. [53]	2006	Head and neck cancer patients receiving conventional or hyperfractionated radiotherapy	11 patients; 5 controls	UPSIT Taste detection thresholds	There were no differences between the patient and control groups for smell and taste sensitivity. There were no significant differences between conventional vs. hyperfractionated courses of radiotherapy. There were significant increases at the 1-month thresholds compared to baseline for all four taste qualities.
Sapir et al. [54]	2016	Stage III and IV oropharyngeal cancer patients	73 patients	H&NQoL instrument UWQoL questionnaire	Significant associations were found between patient-reported severe dysgeusia and radiation dose to the oral cavity and tongue. Salivary output was not significantly correlated with severe taste dysfunction. Xerostomia while eating scores were correlated with severe dysgeusia. Taste scores worsened at 1 month compared to pre-treatment.
Stieb et al. [55]	2022	Oropharyngeal cancer patients	116 patients	MDASI-HN module	MDASI-HN scores for taste impairment significantly correlated with dry mouth scores. Patients with moderate-to-severe taste impairment had significantly more pathological lymph nodes. There was no correlation between the mean and minimum dose of radiation to the ipsilateral parotid gland with moderate-to-severe taste impairment.
Stieb et al. [56]	2020	Oropharyngeal cancer patients	326 patients	MDASI-HN module	Taste improved in the years after completing radiation therapy but plateaued after year 5.
Tharakan et al. [18]	2023	Oropharyngeal cancer patients treated with transoral robotic surgery (TORS)	37 TORS patients; 32 controls	NIH toolbox olfaction scores Whole-mouth taste testing	NIH toolbox scores were similar across study groups at baseline and follow-up. Of the patients, 42% developed new taste disorders at follow-up, while no controls did. In addition, 36% of patients had new persistent bad taste, while no controls did. Patient-reported taste changes after TORS were frequent. TORS patients had decreased taste identification for the tumor side.
Veyseller et al. [57]	2014	Nasopharyngeal cancer patients	24 patients; 14 controls	MRI coronal, axial, and sagittal slices Connecticut Chemosensory Clinical Research Center Test	There was a significant difference in olfactory scores between groups. Olfactory bulb volumes of the healthy control group were significantly higher than the patient group.
Yamashita et al. [19]	2009	Head and neck cancer patients receiving radiotherapy	52 patients	Whole-mouth taste test	The sensitivity of taste declined significantly between the start of testing and the third week after beginning radiotherapy. From the 8th week after radiotherapy began, the sensitivity of taste improved significantly.

## Data Availability

All data, code, and materials used in the analysis are available to any researcher for purposes of reproducing or extending the analysis via institutional materials transfer agreements (MTAs).
